# AI-Supported Functional Pain Phenotyping in Low Back Pain Using Sensor-Based Gait and Neuromuscular Biomarkers

**DOI:** 10.3390/s26165178

**Published:** 2026-08-15

**Authors:** Jan Jens Koltermann, Philipp Floessel, Freya Charlotte Wunderlich, Jil-Justin Funke, Hannes Kaplick, Alexander C. Disch

**Affiliations:** 1 University Comprehensive Spine Center, University Center for Orthopaedics, Trauma and Plastic Surgery, Faculty of Medicine Carl Gustav Carus, Technische Universität Dresden, Fetscherstrasse 74, 01307 Dresden, Germany; jan.koltermann@mailbox.tu-dresden.de (J.J.K.);; 2 Section Sports Medicine and Rehabilitation, University Center for Orthopaedics, Trauma and Plastic Surgery, Faculty of Medicine Carl Gustav Carus, Technische Universität Dresden, Fetscherstrasse 74, 01307 Dresden, Germany

**Keywords:** low back pain, digital biomarkers, wearables, gait analysis, functional phenotyping, artificial intelligence, machine learning

## Abstract

Low back pain (LBP) is associated with altered motor control that may be reflected in sensor-derived features. This exploratory development study analysed 3567 gait-analysis variables from 36 participants using a 15-feature pipeline and leave-one-subject-out evaluation. The primary outcome contrasted pain-free with high pain intensity after 11 participants in the intermediate mild-pain stratum were excluded by design (25 participants; 50 trials). Stacking achieved an accuracy of 0.780 (95% CI 0.620–0.920) and balanced accuracy of 0.731 (95% CI 0.573–0.893). Recurrent features involved the feet, left thigh and multifidus-specific EMG asymmetry. An exploratory two-cluster analysis showed clinical associations but weak separation (silhouette 0.172). As model configuration and comparison were developed within the available cohort, the estimates represent internal exploratory performance rather than validation of a clinical prediction model. The findings are hypothesis-generating and require independent external validation.

## 1. Introduction

Low back pain (LBP) remains one of the major global challenges for contemporary health-care systems. Beyond its clinical burden, LBP carries substantial health-economic relevance, particularly through sickness absence, productivity losses and premature retirement [1,2,3]. Importantly, a comparatively small proportion of people with chronic or recurrent symptoms accounts for a disproportionate share of societal costs [2]. Early identification of functional risk constellations and prevention of recurrent or chronifying trajectories are therefore of considerable clinical and economic importance.

A central component in the pathophysiology and chronicity of LBP is altered motor control during activities of daily living, particularly walking. Meta-analyses and systematic reviews consistently report slower gait speed, shorter step length, altered thoraco-lumbopelvic coordination and reduced lumbar range of motion in persistent or chronic LBP [4,5,6]. Reviews of trunk muscle activity further describe increased paraspinal activation and comparatively stiff movement strategies [7,8,9]. Although not every distal gait marker is altered consistently across cohorts, individual gait studies support this general direction [10,11]. These functional deficits have been linked to maladaptive neuromuscular control of the deep trunk-stabilising musculature. In particular, the lumbar multifidus has been associated with altered recruitment, structural change and functional impairment, whereas altered or compensatory activation has also been described for the more superficial paraspinal musculature, especially the erector spinae [12,13,14,15,16]. Structural, MRI-based and sonographic work further indicates that muscle quality and fatty infiltration of deeper multifidus regions are more closely related to chronicity, activity level and functional adaptation than to a single cross-sectional pain-intensity score [17,18,19,20,21,22,23].

Because visual assessment alone provides only limited insight into the underlying functional and structural alterations, instrumented gait analysis using wearable sensors is attractive for identifying compensatory gait strategies. Floessel et al., for example, showed that the combination of electromyography (EMG) and inertial measurement units (IMUs) enables objective quantification of kinematic markers and neuromuscular patterns in LBP under dynamic, clinically relevant conditions [24,25]. The combined use of both technologies is particularly relevant in LBP because impaired lumbopelvic control may induce compensatory gait patterns at the interface between trunk and lower extremity. Existing studies using comparable set-ups have reported reduced trunk motion, altered pelvis–thorax coordination, changes in step-to-step dynamics and modified paraspinal activation [4,5,6,7,8,24]. Thus, sensor-based assessment is not merely a technical representation of movement but a means of capturing clinically meaningful control and loading patterns that structural imaging may not fully explain. Reviews of functional biomechanical spine assessment further indicate that the field is moving from validation of individual sensors towards multimodal wearable set-ups and algorithm-assisted decision logic under more ecological loading conditions [25,26,27,28,29]. Portable inertial systems and flexible sensor technologies also suggest that lumbopelvic movement patterns can be objectified outside highly specialised motion laboratories [30,31]. Wearable systems therefore create the methodological basis for deriving clinically interpretable digital biomarkers from raw sensor signals, provided that their context of use is clearly defined and the resulting feature space is fit for purpose [25,32].

At the same time, sensor-based diagnostics generate high-dimensional data with many correlated and potentially non-linear variables. Conventional statistical approaches rapidly reach their limits in such feature spaces, whereas machine-learning methods can support pattern recognition, feature selection and automated classification in complex biomedical data [25,33,34]. Initial work suggests that functional abnormalities in LBP can indeed be detected by AI-supported and sensor-based analysis pipelines [24,33,35,36]. The incremental contribution of the present work is not a new sensor modality or an already validated classifier. It lies in comparing clinically defined pain-intensity contrasts, combining distal gait dynamics with neuromuscular markers and examining whether the resulting candidate feature space supports an explicitly exploratory functional interpretation in LBP. The potential value of such approaches lies not only in numerical classification but also in identifying latent functional patterns reflecting altered muscle activation, protective gait strategies, reduced movement amplitudes or lumbopelvic coordination deficits. Such patterns may be clinically relevant because they can become functionally apparent before a clear structural lesion or a stable chronic pain state is established. Furthermore, studies on clinical subgroups, risk profiles and gait-based phenotypes suggest that functional extreme contrasts and composite clinical profiles may be more tractable than a fine-grained ordinal representation of pain stages [33,34,37,38,39]. The difficulty of separating intermediate classes is also supported by work demonstrating pronounced inter-individual heterogeneity in lumbar kinematics and multiple functional movement profiles within clinically similar LBP cohorts [38,39,40]. A phenotypic approach that captures functional loading and control patterns may therefore be more plausible than expecting a strictly linear mapping of ordinal pain intensity.

Against this background, the present study explored whether clinically anchored, gait-derived digital biomarkers may support functional pain phenotyping in LBP. The primary context of use was defined as the distinction between pain-free individuals and participants with high pain intensity, as this target definition most closely resembles a functional triage and risk-stratification scenario. The broader definition of pain-free versus any pain and the three-class setting were deliberately treated as secondary and sensitivity analyses to assess the robustness of the primary observation under more heterogeneous label structures. In an additional exploratory analysis, we examined whether recurrent sensor features showed any descriptive two-cluster structure at participant level; this analysis was not intended to establish discrete phenotypes. Our working hypothesis was that this pronounced clinical contrast would be associated with a reproducible, but not diagnostic, sensor-derived functional profile involving distal gait dynamics and lumbopelvic neuromuscular control. The aim was not to establish a clinically validated classifier but to generate hypotheses on whether clinically interpretable sensor features can form a digital-biomarker space relevant to functional stratification, triage and, prospectively, earlier detection of unfavourable motor patterns.

## 2. Materials and Methods

### 2.1. Data Basis and Outcome Definitions

The gait-analysis data were available in long format and comprised 1924 rows. These corresponded to 74 individual trials from 37 participants, with 26 iteration-specific sensor or signal channels per trial. For each signal, 22 descriptive and dynamic metrics were available, including central-tendency measures, extrema, area-based measures, variability indices and slope- or derivative-based quantities. The accompanying clinical data comprised 41 participants and included pain-intensity information derived from the Chronic Pain Grade Questionnaire (CPGQ) according to the source protocol, in which pain-intensity scores were stratified into 0–10, 11–29 and >29 [24]. In the present analysis, this information was used as a three-level clustered pain-intensity variable (1 = pain-free, 2 = mild pain intensity, 3 = high pain intensity), together with anthropometric data and functional measures. This coding should therefore be understood as a study-specific pain-intensity grouping derived from Korff questionnaire items, not as the original Korff grade I–IV classification. The thresholds were inherited from the parent-study data definition. The >29 threshold denotes the highest pain-intensity stratum in that protocol and was used to maximise clinical contrast with the pain-free reference group. It has no independently established biomechanical meaning and is neither a diagnostic cut-off nor a validated boundary for extreme functional burden. After harmonisation of participant identifiers and merging of the two data sources, 72 trials from 36 participants entered the analysis; each participant contributed exactly two gait trials. Subsequent long-to-wide transformation yielded 572 gait-derived baseline features per trial before further feature construction and clinically informed feature reduction. The flow was 37 participants in the gait export and 41 in the clinical export, with 36 uniquely matched identifiers; one gait-only and five clinical-only identifiers did not enter the matched cohort. Identifiers were trimmed and hyphens removed before an inner join. No empty participant IDs, outcome values or exported gait-feature cells occurred in the analysed files, and no feature-value imputation was performed. The primary subset subsequently excluded the intermediate group by design, not because of missing data. The available archive did not permit retrospective reconstruction of the reasons for non-matching.

Anthropometric data for the matched overall cohort and the primary subcohort are summarised in Table 1. Sex assignment was available for all 36 matched participants through a separate ID-based mapping file.

Clinically, the three groups were treated as increasingly burdened pain-intensity strata: a pain-free or very low-burden reference group, an intermediate or mild pain group and a high pain intensity group. This structure was used to ask whether gait and neuromuscular features reflect functional loading contrasts relevant to LBP genesis and chronification, rather than to claim that pain intensity itself is objectively measured. In the matched overall cohort, self-reported pain intensity averaged 26.1±16.1 points and disability 12.2±13.8 points. At participant level, the clustered pain-intensity groups comprised 9 pain-free participants, 11 participants with mild pain and 16 participants with high pain intensity. The primary clinical outcome was defined as pain-free versus high pain intensity (1 vs. 3). Only participants assigned to clustered pain-intensity group 1 or group 3 were included in this primary analysis, resulting in 25 participants and 50 trials. Excluding the 11 participants in the intermediate stratum reduced label overlap and within-class heterogeneity and was intended to define an extreme-group development contrast. Statistically, this restriction may sharpen the estimated decision boundary and increase apparent discrimination while reducing sample size, precision and applicability to the continuous clinical spectrum. The secondary pain-free-versus-any-pain analysis and the three-class sensitivity analysis were therefore retained to show how performance changed when the intermediate group was included. Thus, the target variable was based on self-reported pain intensity rather than an independent biological or functional reference standard. Model performance was therefore interpreted as evidence of a sensor-detectable functional loading constellation rather than as direct evidence of objective pain-intensity measurement. Pain-free versus any pain (1 vs. 2/3) was analysed as a secondary supervised outcome. The three-class setting (1 vs. 2 vs. 3) was used exclusively as a sensitivity analysis to assess the impact of a more heterogeneous clinical label structure; it was not interpreted as true phenotyping. The exploratory two-cluster analysis was conducted separately and unsupervised, based on recurrent sensor features. Age was not reported because it was not consistently available in the linked source data used for the present analysis.

### 2.2. Sensor and Functional Reference Framework

The methodological interpretation of the sensor configuration was guided by the multisensor set-up described by Floessel et al. [24]. That protocol used five EMG sensors and four IMUs. EMG was recorded bilaterally over the lumbar multifidus and the longissimus thoracis. Two trunk IMUs were positioned at L3 and S1, and two further IMUs were attached bilaterally to the thighs approximately 15 cm above the knee joint line. EMG and IMU signals in the parent study were acquired using a wireless Cometa Mini Wave Infinity system (Cometa S.r.l., Milan, Italy) and EMG and Motion Tools, version 7 (Cometa S.r.l., Milan, Italy). The parent-study acquisition and feature-processing environment was integrated with MATLAB 2024 (The MathWorks, Inc., Natick, MA, USA) [24]. The present secondary analysis did not reacquire or preprocess raw signals; it analysed the derived trial-level feature export using R version 4.5.2 (R Foundation for Statistical Computing, Vienna, Austria). Under standardised conditions, the parent protocol addressed fatigue resistance, mobility and neuromuscular control, lumbopelvic stability and global trunk-muscle function [24]. Although the present local analysis focused on gait-derived features, anatomical and functional interpretation of the resulting predictors was informed by this broader sensor-based framework and by previous work on multifidus activation, trunk stabilisation and pelvic asymmetry in LBP [12,13,41,42,43,44].

According to the parent-study protocol, gait data were acquired during a standardised 100 m walking test. Participants were instructed to cover the route at a brisk, self-selected pace and wore their usual outdoor shoes. IMU and EMG signals were recorded continuously throughout the task. The protocol was intended to capture gait-related fatigue resistance, neuromuscular control and lumbopelvic stability under a functional dynamic condition.

**Figure 1:**
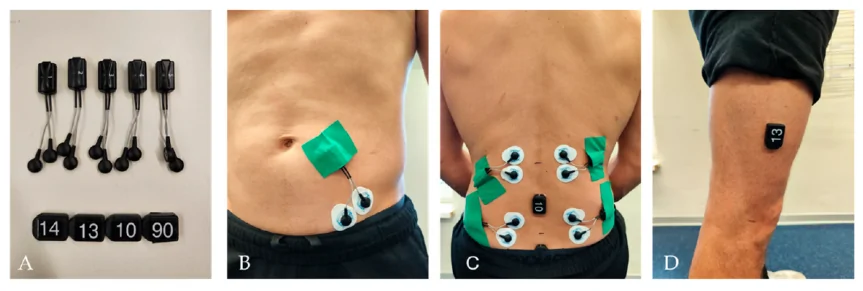
Sensor placement in the reference protocol by Floessel et al. [24]. (**A**) Five surface-EMG sensors (upper row) and four triaxial IMUs (lower row) used in the multisensor protocol. (**B**) Surface-EMG placement over the right internal oblique in the lower abdominal region adjacent to the anterior superior iliac spine. (**C**) Posterior placement of bilateral surface-EMG electrodes over the longissimus thoracis and lumbar multifidus, together with trunk IMUs at the L3 and S1 levels. (**D**) Thigh IMU positioned approximately 15 cm proximal to the knee joint line; placement was mirrored bilaterally.

Figure 1 shows the sensor placement according to Floessel et al. [24]. The original set-up combined paraspinal EMG recordings with trunk and proximal lower-limb IMUs to quantify muscle activation, lumbopelvic control and lower-extremity movement behaviour. The same anatomical logic was used to interpret gait-derived features in the present comparative analysis.

The present analysis used the already derived, trial-level feature export. Raw sensor time series and a complete preprocessing log, including filter settings, cut-off frequencies, segmentation boundaries and window lengths, were not available in the analytical archive. These technical details therefore cannot be reconstructed retrospectively and are not inferred from feature names in this manuscript.

### 2.3. Processing Pipeline

**Figure 2:**
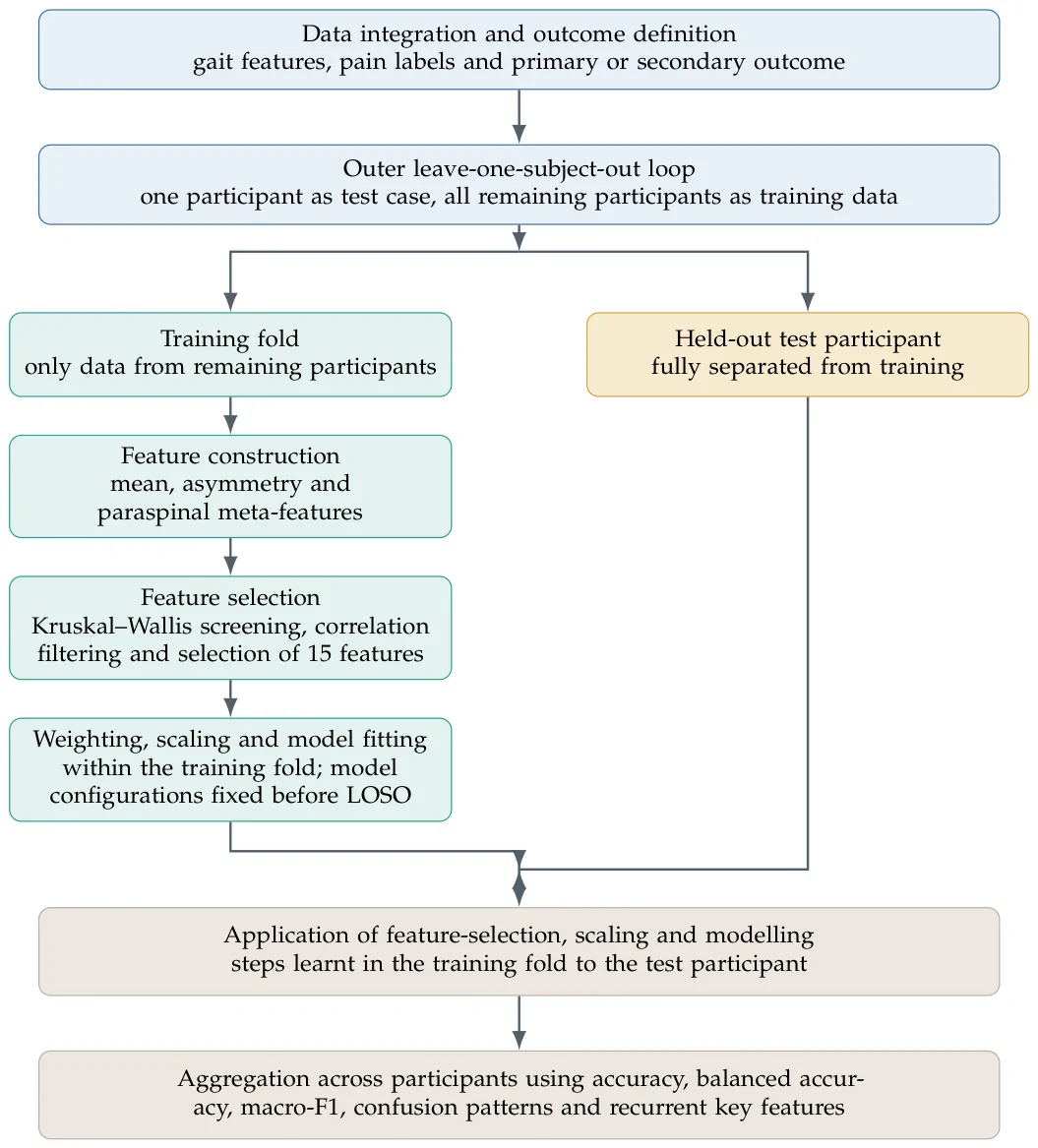
Processing pipeline of the present analysis. In the outer loop, one participant was held out completely. Feature construction, feature reduction, weighting and scaling were estimated exclusively within the training fold and subsequently applied to the held-out participant. Model configurations were selected during development on the available cohort and then held fixed throughout LOSO evaluation.

The analytical workflow is summarised in Figure 2. The same pipeline was applied to the primary and secondary outcomes to ensure direct comparability of model performance. Data preparation and modelling were conducted in R 4.5.2. Data import, reshaping, merging and result export were performed mainly using functions from base, utils and stats; specialised external packages were used primarily for modelling. The R packages and base modules used in the analysis are listed in Appendix A Table A3.

Iteration-specific gait variables were transformed into a wide feature representation, such that each participant–trial combination was represented by a single feature row. The exported metrics were already aggregated per signal and iteration or segment and included not only measures of location and dispersion but also dynamic descriptors. Operationally, max_slope denotes the steepest positive signal increase within the analysed window, max_negative_slope the steepest negative signal decrease, and v_dt a derivative-based feature of temporal signal change. std represents intra-segment variability, rms the effective or energy-related signal amplitude, and Avg/Asym mean or asymmetry parameters derived from left–right feature pairs. The English display term max_negative_slope corresponds to the German identifier max_gefaelle in the original export. The recurrent dynamic features were therefore not created only within the local classification pipeline; they were exported from the original sensor trajectories in the source data and subsequently condensed biomechanically. The pipeline comprised eight steps:

1. **Data integration and outcome definition.** Gait features and questionnaire data were merged after harmonisation of participant identifiers. The primary outcome was defined as pain-free versus high pain intensity; broader pain definitions were analysed as secondary and sensitivity outcomes using the identical processing pipeline.
2. **Feature construction.** In addition to the original gait features, biomechanically motivated meta-features were constructed by combining left and right feature pairs into mean and asymmetry parameters. Bilateral paraspinal meta-features were generated for multifidus and thoracic erector-spinae channels to capture averaged and asymmetry-related EMG signals. This step aimed to better represent inter-side imbalance and compensatory control [42,43,44].
3. **Univariate screening.** Within each training fold, features were initially ranked using the Kruskal–Wallis test. This non-parametric approach allowed robust prioritisation of potentially informative variables without assuming a specific model family.
4. **Correlation-based feature reduction.** After ranking, highly redundant variables were removed using a correlation filter. In the clinically anchored primary analysis, up to two slots were reserved for statistically salient multifidus features, provided they met the screening threshold and were not highly correlated with already selected variables. In total, 15 features were used per fold. The 15-feature ceiling was a pragmatic dimensionality-reduction constraint and should not be interpreted as satisfying a conventional events-per-variable rule; with 25 participants in the primary contrast, the feature-to-participant ratio remains a major source of instability.
5. **Scaling and training-based preprocessing.** Centring and scaling parameters were estimated exclusively from the training data in each fold and then applied to the held-out participant, preventing information leakage from the test data into preprocessing.
6. **Handling of class imbalance.** Because the binary outcomes were imbalanced, inverse class frequencies were used as weights whenever the respective model supported weighted fitting. In addition to accuracy, balanced accuracy and macro-F1 were reported.
7. **Model fitting and model development.** Candidate configurations were compared by five-fold subject-grouped cross-validation on the available analytical cohort before the LOSO model comparison. Decision-tree candidates covered complexity parameters of 0.0005–0.02, minimum split sizes of 4–10 and maximum depths of 6–12; kNN used k=1,3,5,7,9,11,13. LASSO compared lambda.min with lambda.1se; Elastic Net additionally varied α from 0.1 to 0.9. SVM candidates used costs of 0.25–4 and four feature-dimension-dependent gamma values. XGBoost candidates used depths of 2–4, learning rates of 0.03–0.10 and 60–120 boosting rounds. Candidate selection prioritised balanced accuracy, followed by macro-F1 and accuracy. For the primary clinically anchored analysis, the selected configurations were: decision tree cp=0.0005, minimum split =4, maximum depth =6; kNN k=5; LASSO α=1 with lambda.min; Elastic Net α=0.1 with lambda.min; SVM cost =2 and gamma =0.0167; and XGBoost maximum depth =3, learning rate =0.05, 80 boosting rounds, minimum child weight =1, subsampling =0.8 and column subsampling =0.8. These configurations were then held fixed while models were fitted within each LOSO training fold. The compared models were multinomial regression, LASSO, Elastic Net, LDA, decision tree, kNN, SVM, XGBoost, a voting ensemble and a stacking model. Because configuration selection preceded and used the same analytical cohort as LOSO evaluation, this workflow represents exploratory internal model development rather than fully nested internal validation.
8. **Evaluation and digital-biomarker interpretation.** Final model performance was assessed using leave-one-subject-out cross-validation. For model-agnostic interpretability, permutation importance was computed for all models and condensed into top-25 lists and cross-model key-feature summaries. Recurrent features were interpreted as candidates for a clinically anchored digital-biomarker profile. Given the small sample size, these candidates were evaluated descriptively and as hypothesis-generating; formal external stability testing of feature selection was not possible with the available data. The stored LOSO feature log contained the number and top-ranked feature for each fold, but not every member of each 15-feature set; consequently, full fold-wise selection frequencies and confidence intervals for individual feature importance could not be reconstructed.

This pipeline pursued three methodological aims: first, fair comparison of different clinical outcome definitions; second, reduction in redundancy and instability in a relatively small data set; and third, preservation of biomechanical interpretability in the final feature space. Emphasis on asymmetry, lumbopelvic coordination and paraspinal control followed directly from the literature on impaired lumbar stabilisation, altered recruitment, modified gait coordination and pelvic asymmetry in LBP [4,6,8,12,13,41,45]. Selection of multifidus-related and paraspinal features was likewise motivated by literature on structural muscle change, deep multifidus regions and heterogeneous movement phenotypes [17,18,19,38,39,40]. A concise overview of the naming logic and dynamic feature components is provided in Appendix A Table A2.

The model families were selected to cover complementary statistical assumptions. Multinomial regression, LASSO and Elastic Net represented linear or sparsity-oriented baselines; LDA provided a compact linear discriminant model; kNN captured local distance-based neighbourhood structure; decision tree and XGBoost addressed non-linear thresholds and interactions; SVM tested margin-based separation in a potentially high-dimensional feature space; and voting and stacking ensembles were added to evaluate consensus and meta-learning solutions across model families. For a small, correlated and biomechanically heterogeneous data set, this breadth of models allowed interpretable linear and more flexible non-linear decision boundaries to be evaluated under identical data conditions. The comparison was exploratory and did not constitute an a priori test of the superiority of any single algorithm.

### 2.4. Exploratory Cluster Analysis

In addition to supervised modelling, an exploratory, hypothesis-generating unsupervised two-cluster analysis was performed at participant level. For this purpose, the two trials per participant were averaged and 12 stable sensor features were used, comprising 10 cross-model recurrent gait-derived features and the two multifidus-specific asymmetry markers rmsAsym.R_Multifidii and v_dtAsym.R_Multifidii. After z-standardisation, a *k*-means solution with k=2 was computed. Cluster stability was assessed using mean silhouette and repeated subsampling with consensus values. Trial-to-trial reproducibility was evaluated by assigning the two individual trials of each participant to the participant-level centroids and reporting percentage agreement and Cohen’s κ. For clinical anchoring, associations of the resulting cluster labels with clustered pain intensity, disability and continuous pain intensity were examined; the first principal component was additionally explored as a continuous feature-space score. The full clustering call, including its random initialisation settings, was not retained in the available analytical archive and therefore cannot be reported retrospectively. Accordingly, the clustering results are treated only as descriptive internal structure and not as evidence of reproducible biological subgroups.

### 2.5. Ethics and Consent

The underlying research project was approved by the Ethics Committee of Dresden University of Technology (approval number BO-EK-215052022_1). The study was conducted in accordance with the Declaration of Helsinki. All participants received detailed information about study procedures and data-protection requirements before enrolment and provided written informed consent prior to participation.

## 3. Results

### 3.1. Primary Analysis: Pain-Free Versus High Pain Intensity

**Figure 3:**
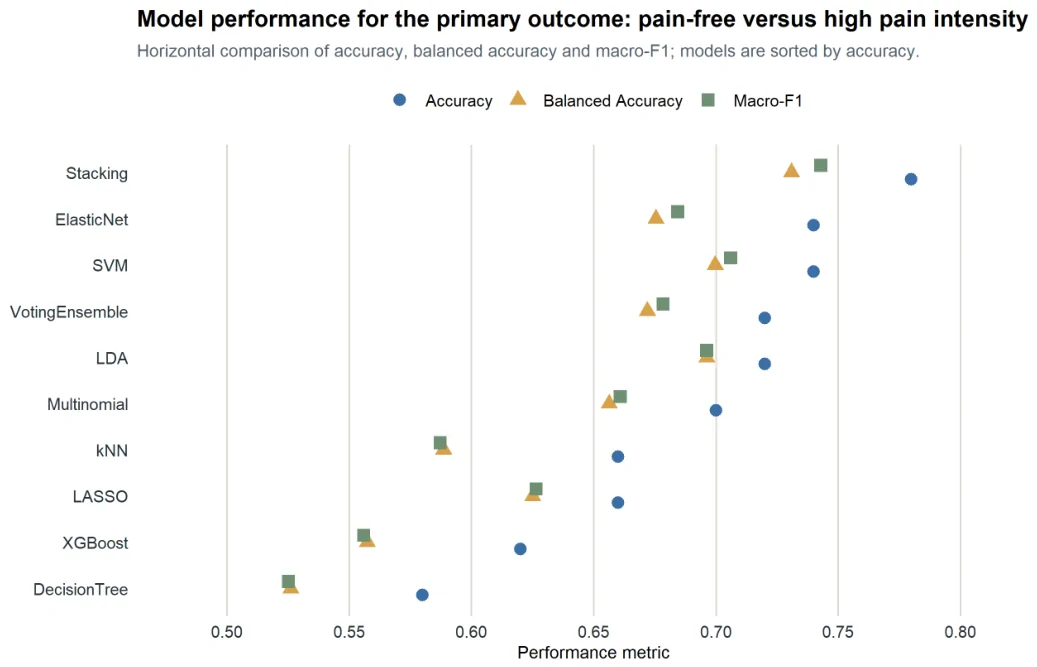
Model comparison for the primary outcome pain-free versus high pain intensity (1 vs. 3). Accuracy, balanced accuracy and macro-F1 are shown for all tested model families.

This primary analysis tested whether the feature space could separate a clinically low-burden reference state from a clearly symptomatic high-burden state, a contrast most relevant to functional stratification rather than adjacent-stage grading. In the primary setting of pain-free versus high pain intensity, the models showed their highest discriminative performance. The best-performing model was stacking, with an accuracy of 0.780 and a balanced accuracy of 0.731; SVM, LDA and Elastic Net followed moderately behind. Thus, the distinction of a pronounced functional pain contrast appeared to be the most plausible use case for the present pipeline. Based on the original subject-level LOSO predictions, the stacking model yielded 95% confidence intervals of 0.620 to 0.920 for accuracy, 0.573 to 0.893 for balanced accuracy and 0.559 to 0.894 for macro-F1. In an additional probabilistic repeat run of the same clinically anchored stacking configuration, threshold-independent metrics were ROC-AUC = 0.666 and PR-AUC = 0.737. Detailed model results are provided in Table 2 and Figure 3. These intervals describe sampling uncertainty in the available participant-level predictions; they do not incorporate uncertainty introduced by data-driven model-configuration selection or comparison across multiple algorithms. The stacking result is therefore retained as the leading exploratory result of the present development analysis, not as an independently validated estimate of clinical performance.

The cross-model feature structure was also clearer in the primary outcome than in the broader target definitions. Recurrent predictors involved the left foot, left thigh, right-foot dynamics and multifidus-specific EMG signals. The features rmsAsym.R_Multifidii and v_dtAsym.R_Multifidii were particularly recurrent. The frequent dynamic descriptors were mainly slope- and derivative-based: max_slope describes the steepest positive signal increase, max_negative_slope the strongest decline, v_dt the velocity of temporal signal change, and std or rms intra-segment variability or effective signal amplitude. The resulting digital-biomarker profile therefore points to a coupled pattern of distal movement dynamics, lumbopelvic control and paraspinal stabilisation. Cross-model recurrence is reported as a descriptive prioritisation criterion and must not be equated with independently established feature-selection stability.

### 3.2. Exploratory Two-Cluster Analysis

**Figure 4:**
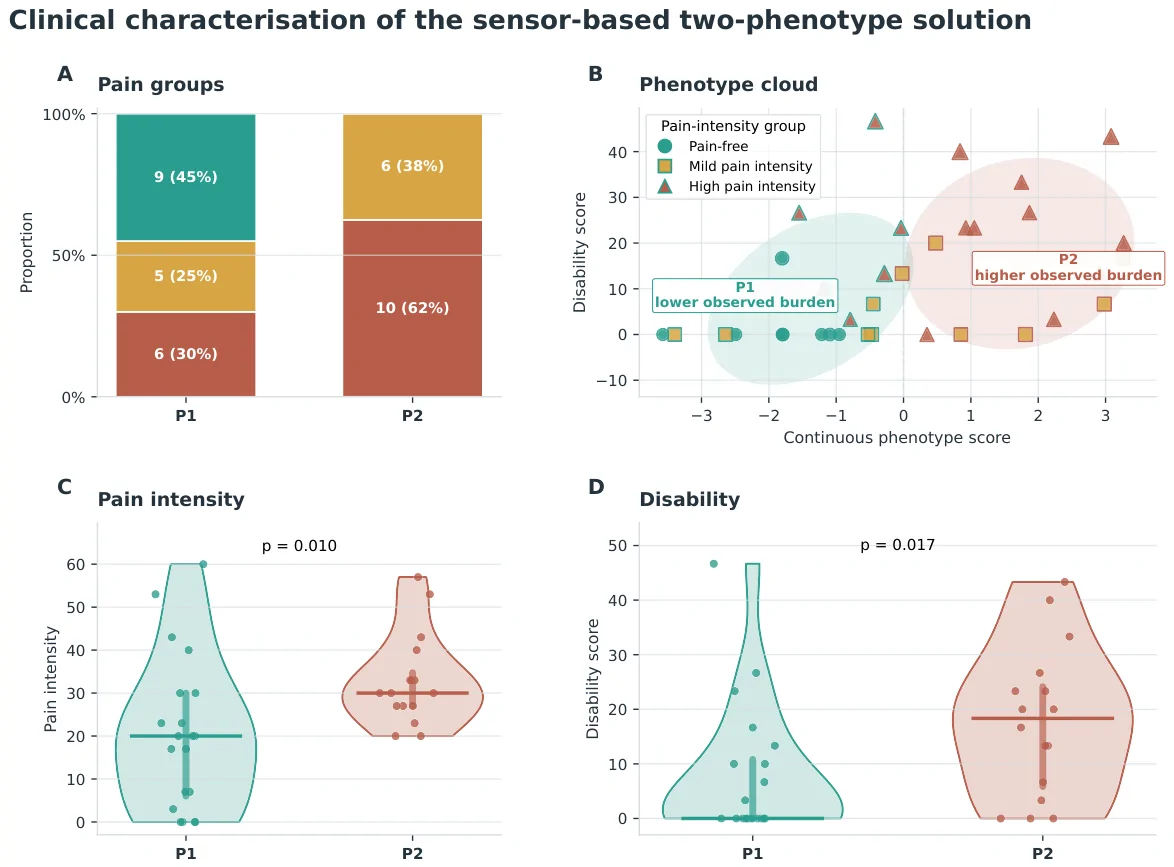
Clinical characterisation of the exploratory two-cluster partition. Panel (**A**) shows the distribution of pain-intensity groups across P1 and P2. Panel (**B**) visualises the participant-level feature cloud using the continuous PC1 score and disability; transparent ellipses are descriptive and do not imply distinct or externally validated cluster boundaries. Panels (**C**,**D**) show pain intensity and disability at participant level.

The exploratory unsupervised participant-level analysis yielded a weakly separated two-cluster partition. The mean silhouette was 0.172, indicating limited separation in feature space; at the same time, within-cluster consensus was high (0.928) and between-cluster consensus was low (0.092). The two trials of each participant received the same cluster label in all cases (agreement = 1.000; Cohen’s κ=1.000). The P1 and P2 labels were associated with clustered pain intensity (Fisher p=0.0048), disability (Kruskal–Wallis p=0.017) and continuous pain intensity (p=0.010). Clinical characteristics are summarised in Table 3 and Figure 4. These findings describe sample-specific partitions within a largely overlapping feature continuum and do not demonstrate discrete functional or biological subgroups. In particular, the high consensus values do not compensate for the weak geometric separation indicated by the silhouette coefficient.

P1 included a relevant proportion of pain-free participants and showed lower disability and pain intensity, whereas P2 contained no pain-free participants and showed higher observed clinical burden. Given the low silhouette, these labels should be understood as a descriptive partition of overlapping functional variation in the present sample, not as established biological subtypes or distinct loading profiles. The analysis therefore provides only a tentative link to the conceptual direction of movement-based subgrouping and phenotyping studies [37,38,39,46,47,48,49].

**Figure 5:**
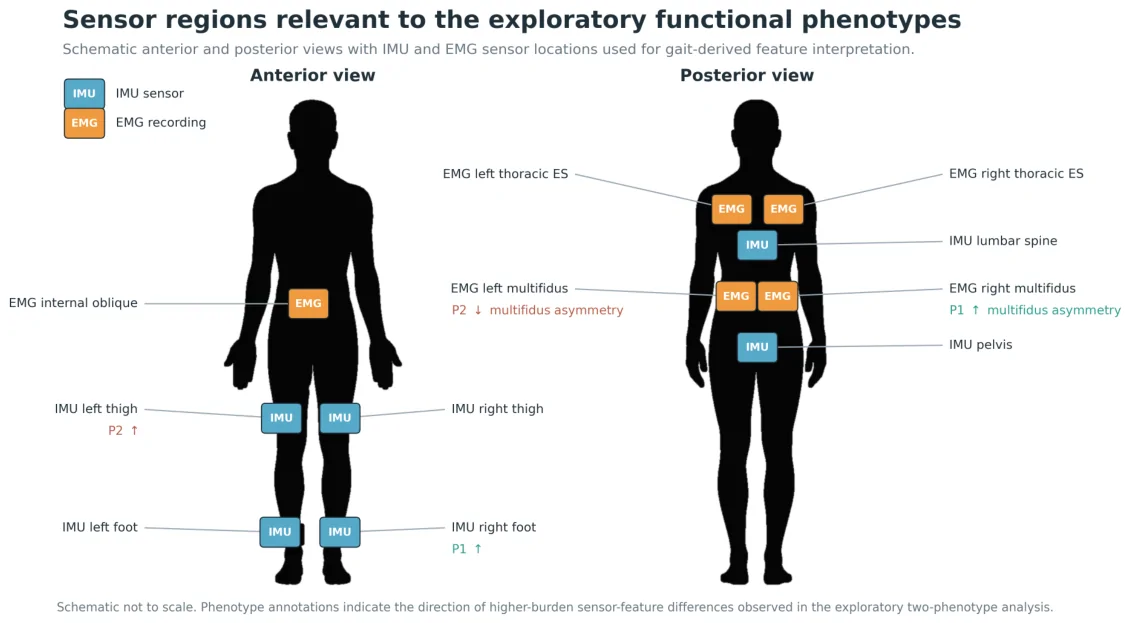
Anatomical localisation of signal regions contributing to the exploratory two-cluster partition. Multifidus-specific asymmetry markers are highlighted in the posterior view, and dynamic markers of the left thigh and right foot in the anterior view. Green annotations indicate feature values that were higher in P1, whereas red annotations indicate feature values characterising P2. Upward and downward arrows denote the direction of the respective feature value relative to the other partition.

As shown in Table 4 and Figure 5, the descriptive differences between P1 and P2 involved mainly dynamic features of the left thigh and right foot and multifidus-specific asymmetry markers. Compared with P1, P2 showed higher right-foot acceleration dynamics, larger left-thigh acceleration changes and more pronounced multifidus-specific EMG asymmetry. The two labels therefore differed descriptively in a coupled distal–proximal sensor pattern rather than in static signal levels alone; owing to the weak overall separation, these differences should not be interpreted as defining distinct biomechanical entities. The continuous phenotype score (PC1) correlated moderately with disability (ρ=0.508; p=0.0016) and clustered pain intensity (ρ=0.581; p=0.0002).

**Figure 6:**
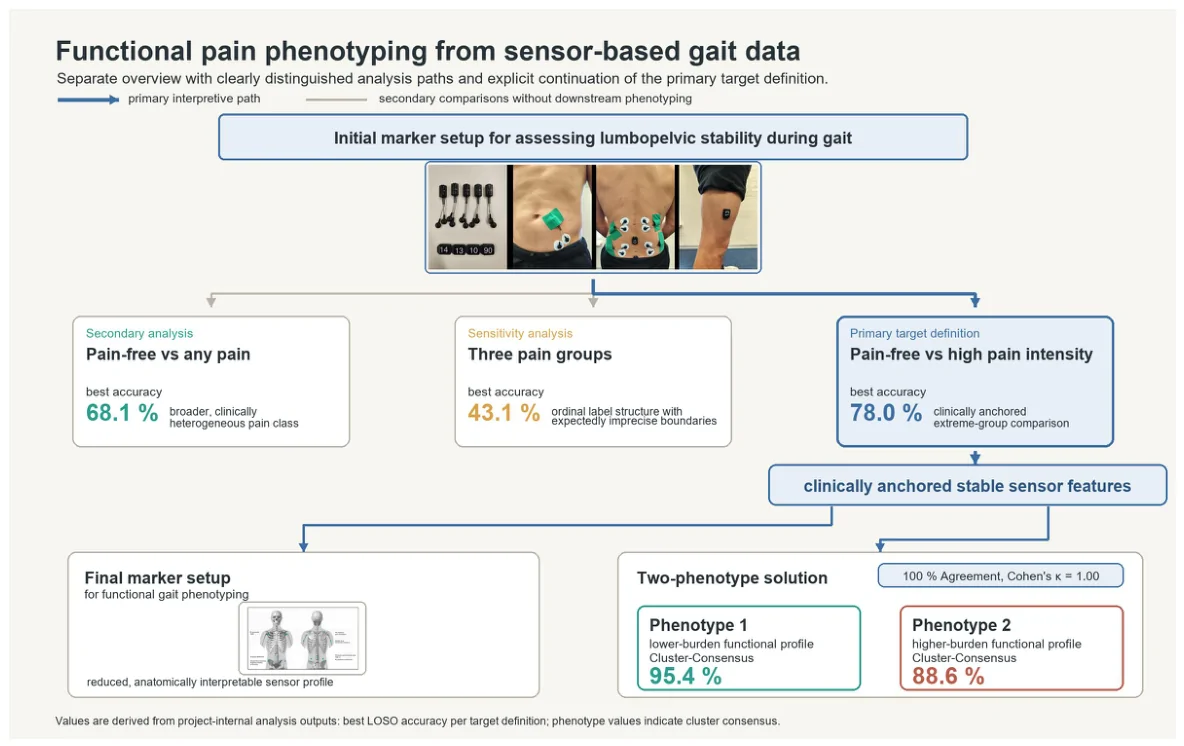
Schematic overview of the analytical and interpretive logic. Starting from the full marker setup, different clinically anchored target definitions were modelled; the primary setting, pain-free versus high pain intensity, achieved the highest accuracy. The resulting stable sensor features were subsequently condensed into an exploratory two-phenotype solution.

Overall, Figure 6 illustrates that the highest model performance was achieved for the clinically clearly defined extreme-group comparison between pain-free participants and participants with high pain intensity. The exploratory cluster partition was therefore derived from stable features of this primary analysis rather than from the more heterogeneous secondary outcome definitions.

### 3.3. Secondary and Sensitivity Analyses

**Figure 7:**
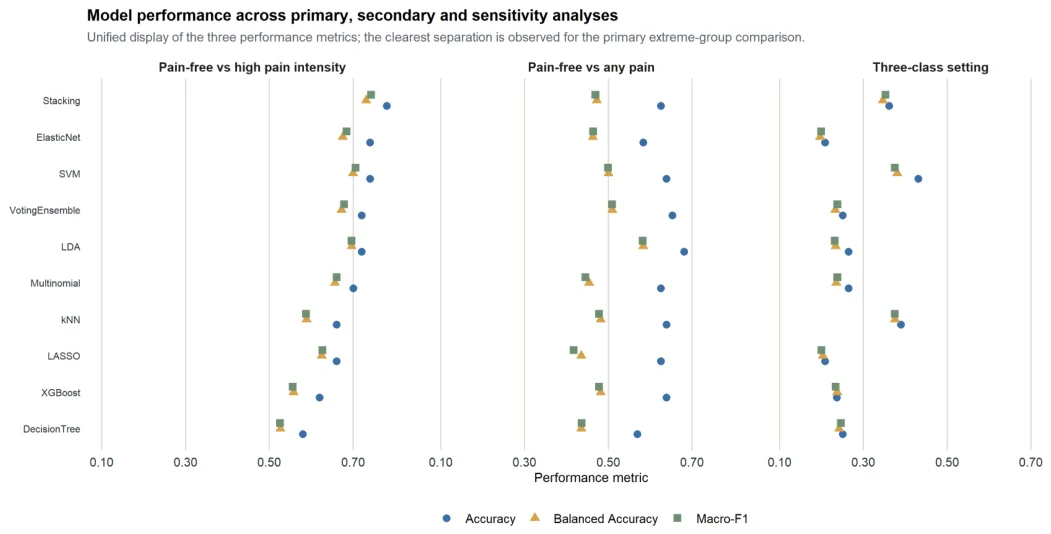
Integrated model comparison across the primary, secondary and sensitivity analyses. Accuracy, balanced accuracy and macro-F1 are shown for the primary outcome pain-free versus high pain intensity, the secondary analysis pain-free versus any pain and the three-class setting.

The secondary analysis tested whether the same feature space remained informative when mild and high pain intensity were collapsed into one broader pain-positive group. In the secondary setting of pain-free versus any pain, discriminative performance remained moderate. LDA achieved the highest balanced accuracy (0.583) with an accuracy of 0.681 (Table 5). Thus, the broader definition of a pain-positive class was more separable than the three-class setting, but remained clearly below the primary extreme-group comparison. The integrated model overview in Figure 7 illustrates this gradation across all three target definitions.

The three-class sensitivity analysis examined whether the sensor features supported a finer ordinal separation of all three clinical strata. In the three-class setting, discriminative performance remained limited. SVM achieved the highest accuracy (0.431) and balanced accuracy (0.380), closely followed by kNN (Table 6). Simultaneously separating pain-free participants, participants with mild pain and participants with high pain intensity therefore remained difficult despite reduction to 15 features. This analysis was consequently interpreted as a sensitivity test for a more heterogeneous clinical label structure.

The sensitivity analysis for the primary outcome showed that a performance-oriented variant without reserved multifidus slots achieved the highest observed model performance, but simultaneously weakened the clinical anchoring of the feature space (Table 7). This revealed a clear trade-off between maximal classification performance and biomechanical interpretability.

Because all model families within a fold were trained on the same clinically anchored 15-feature space, a simple count of identical variables across models is of limited interpretive value. Table 8 therefore summarises the recurrent digital biomarkers in the primary feature space directly in interpretive terms.

## 4. Discussion

The aim of this study was to examine whether digital gait-derived biomarkers can support identification of maladaptive lumbopelvic movement patterns in participants with LBP. The focus was on functional differentiation of pain-related phenotypes. The primary analysis contrasted pain-free participants with participants reporting high pain intensity, whereas secondary and sensitivity analyses contextualised the primary observations. The study should be understood as an exploratory development analysis of a candidate sensor profile; it neither establishes an objective measure of pain nor validates a clinical decision tool.

The results indicate that the clinical outcome definition is central to the suitability of a sensor-based digital-biomarker profile. In the primary outcome, the clinically anchored pipeline achieved the strongest separation, whereas broader or more ordinal outcome definitions yielded substantially weaker performance. Given the small sample size and absence of external validation, however, this observation should not be interpreted as evidence of clinical generalisability, but rather as an exploratory signal. For the proposed context of use, the current model appears more suited to functional phenotyping of pronounced pain contrasts than to a linear three-level representation of pain intensity. Beyond model comparison alone, the study examined whether clinically interpretable sensor features may constitute a digital-biomarker space relevant to stratification, triage and early recognition of unfavourable motor patterns.

This ranking is clinically plausible. The more heterogeneous a target class is, the more likely functional movement patterns are to overlap. In particular, the intermediate pain group probably includes individuals whose gait behaviour is closer either to pain-free participants or to strongly symptomatic participants. Decision boundaries therefore become less distinct and classification more complex. This observation accords with meta-analytic and review evidence indicating more robust signals for altered motor control, lumbopelvic stabilisation, trunk-related activation and biomechanical heterogeneity than for a fine-grained representation of pain intensity [4,5,6,7,9]. Studies on functional subgroups, fear avoidance and inter-individual lumbar-kinematic heterogeneity further show that gait patterns in LBP are influenced not only by reported pain intensity but also by latent control and loading profiles [37,38,39,40,50,51]. The literature therefore supports interpreting the primary outcome as an expression of a pronounced functional contrast rather than as a finely graded pain scale.

The additional two-cluster analysis cautiously supports this interpretation but must be evaluated conservatively. P1 had lower disability and pain-intensity values, whereas P2 contained no pain-free participants; these sample-specific labels summarise overlapping functional variation rather than naturally distinct clinical subgroups. The mean silhouette of 0.172 indicates only weak geometric cluster separation. The complete trial-to-trial agreement therefore primarily indicates high reproducibility of assignment within the present data set; it does not establish clearly separable biological subgroups. The clustering can therefore motivate only a hypothesis about a continuous feature structure and cannot confirm discrete pain phenotypes or loading profiles. This is consistent with movement-based subgrouping work using AI or unsupervised approaches [37,38,39,46,47], and with the broader conceptual framework of biomechanical and pain-mechanistic phenotyping in the BACPAC and BACPAP contexts [28,48,49]. The data do not permit causal differentiation between control models and should be regarded as a starting point for external replication studies.

From a biomechanical and sport-science perspective, the lower right-foot slope, variability and RMS values observed in P1 are compatible with smoother load acceptance and less abrupt rollover, while its lower multifidus asymmetry is compatible with less asymmetric paraspinal recruitment. P2 showed higher right-foot dynamics, stronger left-thigh acceleration changes and greater multifidus-specific asymmetry, a descriptive combination that may reflect altered distal load processing together with lumbopelvic stabilisation demands. These interpretations remain tentative: the weak silhouette does not demonstrate two distinct gait strategies, and the sensor differences may represent positions along a continuous functional spectrum. They may nevertheless inform hypotheses for future studies of gait economy, symmetrical load transfer, pelvic control and deep paraspinal recruitment.

Clinically motivated restrictions and pure performance maximisation were not equivalent. Reserving multifidus slots allowed paraspinal EMG features to remain represented among the final key features. In the primary outcome, rmsAsym.R_Multifidii and v_dtAsym.R_Multifidii recurred as relevant markers, increasing the biological plausibility of the feature space. This observation is supported by multifidus literature describing atrophy, fatty infiltration, deep muscle quality and chronicity-related changes more consistently than a direct mapping to momentary pain intensity [16,17,18,19,20,21,23,52,53,54]. At the same time, intervention literature suggests that morphological improvements in the multifidus need not translate linearly into clinical endpoints [55]. The sensitivity analysis showed that the highest observed balanced accuracy was obtained in a performance-oriented variant without reserved multifidus slots. This gain should therefore be interpreted as a methodological trade-off rather than as a clinically superior pathway. The reserved slots were investigator-guided and introduce potential confirmation bias. Because every model in the anchored branch was fitted after the same constrained preselection, cross-model recurrence of multifidus features is conditional on that design choice and does not constitute independent corroboration. Accordingly, the recurrent biomarkers listed in Table 8 are biomechanically interpretable candidates, not validated or selection-stable markers. Their robustness must be tested by unconstrained feature selection across repeated resamples and subsequently in an independent cohort. The unconstrained sensitivity branch is therefore essential context for interpreting these markers.

The recurrent features support a biomechanical reading of the digital-biomarker profile. Across binary outcome definitions, relevant predictors clustered around the left foot, left thigh, right-foot dynamics, lumbopelvic acceleration regulation and paraspinal asymmetry signals. Slope, variability and asymmetry measures were particularly frequent. This suggests that dynamic control variables, rather than isolated amplitudes, were informative for the models. Such a pattern is compatible with altered load-transfer strategies, compensatory redistribution within the lower-extremity–trunk system and impaired lumbopelvic stabilisation [15,42,43,44]. The repeated relevance of multifidus-related and trunk-related signals also fits the assumption that deep segmental stabilisation and superficial compensation are functionally linked in chronic LBP [13,14].

Methodologically, the findings underline the potential of wearable sensing combined with machine learning. The pipeline extracted recurrent predictors from a high-dimensional, correlated feature space, which is important for digital wearable diagnostics where large data volumes and potentially non-linear relationships are common [25,33,34]. Recent reviews and technological studies indicate that wearable back-sensor research is increasingly oriented towards functional phenotyping, multimodal pattern recognition and objective monitoring [26,27,28,29,56]. In a translational clinical scenario, such a profile may be most useful where functional triage, risk stratification or selection of further assessments is required, rather than as a stand-alone replacement for comprehensive clinical evaluation. Beyond screening, future context-of-use scenarios include preventive monitoring, phenotype-oriented exercise prescription, return-to-activity or return-to-competition decisions, and postoperative load progression after spinal interventions. In each of these settings, the clinically relevant question would not be whether the model assigns a definitive pain label, but whether it identifies a candidate sensor pattern that can guide timing, intensity and type of intervention. These applications are prospective contexts of use only. They require predefined decision thresholds, comparison with conventional clinical assessment, prospective calibration and independent validation before any patient-level implementation.

Several limitations are central to interpretation. First, the sample was small. Although 36 participants with 72 trials entered the overall analysis, the primary analysis was based on only 25 participants and 50 trials. Even subject-wise leave-one-subject-out validation cannot exclude unstable performance estimates at this sample size, and the reported confidence intervals reflect this uncertainty. Second, no external validation in an independent cohort was available. All models, key features and cluster labels were derived from the same data set, leaving transferability to other populations, measurement protocols and clinical settings unresolved. Third, the target variable was based solely on self-reported pain intensity. Because pain is multidimensional and modulated by functional, psychosocial and contextual factors, it remains unclear whether the models captured pain intensity in a narrow sense or rather functional limitations, protective strategies and movement profiles. The results suggest the latter. Fourth, statistical uncertainty analysis was limited. Confidence intervals were reported for the best primary model, but not systematically for all model families or all key features. Similarly, cross-model recurrence of features does not replace formal feature-selection stability analysis across independent samples or extensive resampling. Fifth, the two-cluster analysis was hypothesis-generating. The silhouette of 0.172 indicates only weak cluster separation; high consensus and trial-to-trial values therefore demonstrate a consistent structure in the present data set rather than confirmed biological subgroups. Finally, the analysis used already aggregated gait features and thus captures only part of the functional spectrum of LBP; raw time-series patterns and task-general dynamics remain unaddressed. Probabilistic calibration of the primary stacking model was also weak (Brier score 0.237, calibration slope 0.089, expected calibration error 0.260). A calibration slope far below 1 indicates that the fitted probabilities were excessively extreme and do not provide a reliable probability scale in new participants. The current output must therefore not be used to define individual risk thresholds, estimate positive or negative predictive values, support triage probabilities or guide prospective clinical decisions. At most, the model supports exploratory comparison of discrimination and class assignment within this cohort; recalibration and its prospective evaluation would have to be developed in an independent sample. No non-sensor clinical baseline based on questionnaire or routine clinical variables was fitted; consequently, the present study cannot demonstrate incremental value of the sensor-based models over conventional assessment. Moreover, a single standardised walking test cannot represent task-general motor behaviour across lifting, bending, prolonged loading or other activities relevant to chronic LBP. Model configurations were selected through subject-grouped cross-validation in the full analytical cohort before LOSO fitting. The reported LOSO performance can therefore retain selection optimism and should not be interpreted as a fully nested validation estimate. Exact raw-signal preprocessing settings and complete fold-wise feature sets were not preserved in the analytical archive, limiting reproducibility of the upstream signal-processing and feature-stability analyses. These constraints reinforce the exploratory status of all reported performance and biomarker findings.

In summary, the findings support further development of wearable sensing and machine learning for functional phenotyping in LBP, but explicitly at an exploratory and hypothesis-generating level. The clinically anchored digital-biomarker profile is most plausible and interpretable for the extreme-group comparison of pain-free versus high pain intensity. The most appropriate future use case is therefore less fine-grained pain classification than functional phenotyping, triage and risk stratification, which require further testing in larger, multimodal and externally validated cohorts. A particularly important question is whether the functional profiles described here already exist in pain-free individuals as predisposing control patterns or emerge only during persistent pain. This distinction is likely to be central for prevention, early risk detection and phenotype-oriented intervention. If confirmed externally, such profiles could provide a basis for assigning targeted therapeutic interventions to functional phenotypes rather than relying solely on symptom intensity.

## Figures and Tables

**Table 1:** Anthropometric characteristics of the matched overall cohort and the primary subcohort, overall and stratified by sex.

Variable	Overall	Women	Men
**Matched overall cohort**			
Participants, *n*	36	21	15
Trials, *n*	72	42	30
Height, cm	173.6±8.6	168.3±5.4	180.9±6.6
Body mass, kg	75.5±14.8	68.6±12.0	85.3±13.0
Body mass index, kg/m^2^	24.9±3.5	24.2±3.8	26.0±2.9
**Primary subcohort (pain-free vs. high pain intensity)**			
Participants, *n*	25	14	11
Trials, *n*	50	28	22
Height, cm	174.1±9.1	167.9±4.5	181.9±7.3
Body mass, kg	76.3±15.6	67.8±12.2	87.2±12.5
Body mass index, kg/m^2^	25.0±3.6	24.0±4.1	26.3±2.7

**Table 2:** Model performance for the primary outcome pain-free versus high pain intensity (1 vs. 3), sorted by accuracy.

Model	Accuracy	Balanced Accuracy	Macro-F1
Stacking	0.780	0.731	0.743
SVM	0.740	0.700	0.706
ElasticNet	0.740	0.675	0.684
LDA	0.720	0.696	0.696
VotingEnsemble	0.720	0.672	0.678
Multinomial	0.700	0.656	0.661
LASSO	0.660	0.625	0.626
kNN	0.660	0.589	0.587
XGBoost	0.620	0.557	0.556
DecisionTree	0.580	0.526	0.525

**Table 3:** Clinical characterisation of the exploratory two-cluster partition at participant level.

Cluster Label	*n*	Clustered Pain Intensity 1/2/3	Disability, Mean (Median)	Intensity, Mean (Median)	Descriptive Pattern
P1	25	9/10/6	8.3 (3.3)	21.0 (20.0)	Lower observed clinical burden and a relevant proportion of pain-free participants.
P2	11	0/1/10	21.0 (21.1)	37.7 (40.0)	Higher observed disability and pain intensity; no pain-free participants.

**Table 4:** Differences in sensor features used for the exploratory two-cluster partition at participant level.

Feature	P1 Mean	P2 Mean	Difference P2–P1
rmsAsym.R_Multifidii	0.001	0.006	0.005
v_dtAsym.R_Multifidii	0.000	0.002	0.002
max_slopeR.R_Right_Foot_Acc_Y	0.016	0.032	0.016
max_slopeAvg.R_Right_Foot_Acc_Y	0.014	0.027	0.013
stdR.R_Right_Foot_Acc_Y	0.012	0.023	0.011
rmsL.R_Right_Foot_Acc_Z	0.018	0.030	0.012
max_slopeR.L_Left_Thigh_Acc_Z	0.009	0.017	0.008
max_negative_slopeR.L_Left_Thigh_Acc_Z	−0.011	−0.020	−0.009
minAsym.L_Left_Thigh_Acc_Z	−0.004	−0.010	−0.006
v_dtAvg.L_Left_Thigh_Acc_Z	0.006	0.008	0.002

**Table 5:** Model performance for the secondary analysis pain-free versus any pain (1 vs. 2/3), sorted by accuracy.

Model	Accuracy	Balanced Accuracy	Macro-F1
LDA	0.681	0.583	0.582
VotingEnsemble	0.653	0.509	0.509
SVM	0.639	0.500	0.499
kNN	0.639	0.481	0.478
XGBoost	0.639	0.481	0.478
Stacking	0.625	0.472	0.469
Multinomial	0.625	0.454	0.445
LASSO	0.625	0.435	0.417
ElasticNet	0.583	0.463	0.464
DecisionTree	0.569	0.435	0.436

**Table 6:** Model performance for the three-class sensitivity analysis (1 vs. 2 vs. 3), sorted by accuracy.

Model	Accuracy	Balanced Accuracy	Macro-F1
SVM	0.431	0.380	0.375
kNN	0.389	0.375	0.375
Stacking	0.361	0.346	0.353
Multinomial	0.264	0.234	0.238
LDA	0.264	0.233	0.231
DecisionTree	0.250	0.242	0.246
VotingEnsemble	0.250	0.232	0.238
XGBoost	0.236	0.237	0.234
LASSO	0.208	0.203	0.199
ElasticNet	0.208	0.196	0.199

**Table 7:** Sensitivity analysis for the primary outcome pain-free versus high pain intensity.

Variant	Seed	Best Model	Accuracy	Bal. Acc.	Macro-F1
Clinically anchored primary analysis with reserved multifidus slots	42	Stacking	0.780	0.731	0.743
Performance-oriented analysis without reserved multifidus slots	42	SVM	0.860	0.818	0.836
Performance-oriented analysis without reserved multifidus slots	2026	LDA/Multinomial	0.780	0.755	0.758

**Table 8:** Interpretation of recurrent digital biomarkers in the clinically anchored feature space.

Feature	Interpretation
max_negative_slopeR.L_Left_Thigh_Acc_Z	Maximum negative slope of Z-axis acceleration of the left thigh in the right-referenced segment; describes the steepest signal decline.
max_slopeAvg.R_Right_Foot_Acc_Y	Averaged left–right foot feature describing the steepest positive increase in Y-axis acceleration; the name follows the right-foot variable.
max_slopeR.L_Left_Foot_Acc_Y	Maximum positive increase in Y-axis acceleration of the left foot in the right-referenced segment or analysis window.
max_slopeR.L_Left_Thigh_Acc_Z	Maximum positive increase in Z-axis acceleration of the left thigh in the right-referenced segment; captures rapid changes in proximal leg movement.
minAsym.L_Left_Thigh_Acc_Z	Left–right asymmetry feature based on the minimum Z-axis acceleration of the left thigh; indicates inter-side differences.
rmsAsym.R_Multifidii	Asymmetry feature of the root-mean-square value of the multifidus-specific EMG signal in the right-referenced segment; represents differences in paraspinal activation strength.
rmsL.R_Right_Foot_Acc_Z	Root-mean-square value of Z-axis acceleration of the right foot in the left-referenced segment; represents overall signal amplitude.
stdR.R_Right_Foot_Acc_Y	Standard deviation of Y-axis acceleration of the right foot in the right-referenced segment; a measure of variability in foot movement.
v_dtAvg.L_Left_Thigh_Acc_Z	Averaged derivative-based change feature of Z-axis acceleration of the left thigh; describes the speed of temporal signal change.
v_dtAsym.R_Multifidii	Left–right asymmetry feature of temporal change in multifidus-specific EMG activity; indicates asymmetric recruitment dynamics.

## Data Availability

The derived data underlying the reported analyses and the project-specific analysis code are available from the corresponding author upon reasonable request, subject to applicable data-protection and project requirements. The raw sensor time series and a complete upstream preprocessing log were not part of the analytical archive used for this secondary analysis. Public release of the documented analysis code will be considered after removal of project-specific paths.

## Abbreviations

The following abbreviations are used in this manuscript:

AI	artificial intelligence
CPGQ	Chronic Pain Grade Questionnaire
EMG	electromyography
IMU	inertial measurement unit
LBP	low back pain
LOSO	leave-one-subject-out
RMS	root mean square

## Appendix A. Supplementary Tables

**Table A1:** Compact overview of the supplementary PubMed evidence synthesis.

Evidence Domain	Main Finding	Relevance to the Present Study
Gait and kinematics	Reviews report slower walking, shorter steps, reduced lumbar motion and altered trunk coordination in LBP [4,5,6,10].	Supports gait-derived IMU features as plausible digital biomarkers.
Paraspinal activation	Reviews and functional EMG studies describe altered trunk and paraspinal activation during walking and functional tasks [7,15,57].	Supports paraspinal EMG features as biomechanically plausible without assuming a direct pain scale.
Multifidus structure and quality	Consistent evidence links multifidus atrophy, fatty infiltration and deep muscle quality to chronicity and function [16,17,20,21].	Supports inclusion of multifidus-related features as clinically interpretable markers.
Lumbopelvic control and asymmetry	Studies on pelvic asymmetry, paraspinal asymmetry and muscle quality indicate associations with LBP, symptom duration and compensatory movement strategies [23,42,43,44,53,54].	Explains the relevance of asymmetry-related and lumbopelvic features.
Wearables and digital biomarkers	Reviews, proof-of-concept studies and emerging sensor technologies emphasise the potential of wearable systems for objective, clinically useful digital biomarkers [26,27,28,29,30,31,56].	Justifies positioning the present work as sensor-based digital-biomarker analysis.
Machine learning and latent profiles	Sensor and ML studies indicate more robust classification of functional subgroups, risk profiles and extreme contrasts than of ordinal pain stages [33,35,36,37,38,39,50,51].	Explains why the primary outcome pain-free versus high pain intensity was most separable.

**Table A2:** Glossary of recurrent naming components in the analysed feature space.

Name Component	Meaning
mean	Arithmetic mean of a signal within the respective segment.
std	Standard deviation of a signal as a measure of variability.
var	Variance of a signal within the segment.
rms	Root-mean-square amplitude of a signal.
min	Minimum observed signal value.
max	Maximum observed signal value.
auc	Area under the signal curve over time.
max_slope	Steepest positive signal increase over time.
max_negative_slope	English display label for the original export identifier max_gefaelle; steepest negative signal decrease over time.
v_dt	Derivative-based change feature describing temporal signal dynamics.
Avg	Averaged feature derived from a left–right feature pair.
Asym	Asymmetry feature derived from a left–right feature pair.
L/R	Left- or right-related segment, side tag or summary origin according to export convention.
L_Left_Foot	Sensor or feature of the left foot.
R_Right_Foot	Sensor or feature of the right foot.
L_Left_Thigh	Sensor or feature of the left thigh.
R_Right_Thigh	Sensor or feature of the right thigh.
Pelvis	Pelvis-sensor feature.
LWS	Feature of the lumbar-spine region.
Multifidii	Feature of the EMG channel over the multifidus muscle.
ThoracicEs	Feature of the thoracic erector-spinae or longissimus-related channel according to export naming.
Acc	Acceleration signal.
X, Y, Z	Sensor axis of the respective acceleration signal.

**Table A3:** R packages and base modules used in the analysis.

Package	Role in the Analysis
base	Core data structures, recoding, merging, loop logic and helper functions.
utils	Reading and writing CSV files, especially via read.csv, read.csv2 and write.csv.
stats	Long-to-wide transformation via reshape, univariate tests, correlation analysis, scaling and other statistical base functions.
graphics	Base graphics for bar charts, tree plots, axes and legends.
grDevices	Export of analysis figures as PNG files.
MASS	Linear discriminant analysis (LDA).
nnet	Multinomial regression.
rpart	Decision-tree modelling.
class	*k*-nearest-neighbours classification.
glmnet	Regularised linear models, especially LASSO and Elastic Net.
e1071	Support-vector-machine modelling.
xgboost	Tree-based gradient boosting for non-linear interaction structures.
